# Electrospun Poly(methyl methacrylate)/TiO_2_ Composites for Photocatalytic Water Treatment

**DOI:** 10.3390/polym13223923

**Published:** 2021-11-13

**Authors:** Olya Stoilova, Nevena Manolova, Iliya Rashkov

**Affiliations:** Laboratory of Bioactive Polymers, Institute of Polymers, Bulgarian Academy of Sciences, Akad. G. Bonchev St., bl. 103A, 1113 Sofia, Bulgaria; manolova@polymer.bas.bg (N.M.); rashkov@polymer.bas.bg (I.R.)

**Keywords:** electrospinning, poly(methyl methacrylate), TiO_2_, UV-induced wettability, mechanical properties, photocatalysis

## Abstract

Electrospinning was successfully used for the one-step fabrication of poly(methyl methacrylate) (PMMA) fibers loaded with an inorganic photocatalyst—titanium oxide (TiO_2_). By tuning the PMMA/TiO_2_ ratio and the electrospinning conditions (applied voltage, needle tip-to-collector distance, and flow rates), PMMA/TiO_2_ composites with selected organic/inorganic ratios, tailored designs, and targeted properties were obtained. The morphology of the electrospun composites was affected by the amount of TiO_2_ incorporated into the PMMA fibers. In addition, the inorganic photocatalyst had an impact on the wettability, thermal stability, and optical properties of the electrospun composites. In particular, the surface wettability of the composites was strongly influenced by UV light irradiation and from hydrophobic became superhydrophilic. Moreover, PMMA/TiO_2_ composites had enhanced tensile strength in comparison with those of bare PMMA mats. The electrospun PMMA/TiO_2_ composites showed excellent photocatalytic efficiency against the model organic pollutant—methylene blue—which is very promising for the future development of membranes that are highly efficacious for photocatalytic water treatment.

## 1. Introduction

The creation of cost-effective and advanced materials for water treatment is an ongoing challenge. Among the various new materials that have been created, electrospun membranes are considered to be the most versatile candidates for the effective treatment of water, filtration, and separation because of their high surface area, high porosity, and light weight [1,2]. In order to improve the applicability and performance of electrospun membranes for use in water treatment, metal oxide nanoparticles have been incorporated into membranes [2,3]. Thus, the key challenge in water treatment is to prepare appropriate electrospun composites with a high efficiency and low environmental impact.

Currently, great attention is being paid to photocatalysis, which is one of the most effective and ecofriendly techniques for the degradation of many organic pollutants [4,5,6]. In this regard, one of the most widely reported on and commonly used photocatalysts for the removal of organic pollutants is TiO_2_ because of its ability to generate hydroxyl radicals (HO^•^) and superoxide radical anions (O^2•−^) upon UV light irradiation [7]. Furthermore, TiO_2_ has found a wide range of applications due to its remarkable physical, electronic, dielectric, and optical properties, as well as its efficient photoactivity, high stability, low cost, and friendliness to the environment [7,8,9]. Various studies have revealed that poly(methyl methacrylate) (PMMA) is an appropriate host matrix for these photocatalysts because of its excellent transparency to light, good environmental inertness, chemical and thermal stability, and relatively low cost [10,11,12]. This is why PMMA-based composite films have played a major role in the preparation of light emitting diodes (LED), supercapacitors, optical lenses, sensors, catalysts, etc. Moreover, PMMA/TiO_2_ nanocomposites have remained a hot research topic for use in multifunctional applications, such as photocatalysts, protective coatings, and UV shields [13,14,15,16,17].

Electrospinning has emerged as an appropriate technique for preparing polymer composites loaded with inorganic particles. Although electrospun PMMA/TiO_2_ composites have been recently reported on [18], to the best of our knowledge there has been no thorough study on the effect of the use of TiO_2_ nanoparticles as fillers in uniform defect-free fibers on the properties of electrospun PMMA/TiO_2_ composites. In the past few years, we have demonstrated the possibility of the fabrication of multifunctional hybrid materials by electrospinning [19,20,21]. Electrospun materials based on poly(3-hydroxybutyrate) and TiO_2_ with tailored designs have displayed an excellent stability and been shown to be able to preserve their photocatalytic activity almost completely. In this context, in the present study commercially available TiO_2_ nanoparticles were dispersed into PMMA solution in order to prepare composite PMMA fibers filled with TiO_2_ in one step through electrospinning. By varying the TiO_2_ content (5 and 10 wt. %) in the dispersions and the electrospinning conditions (applied voltage, needle tip-to-collector distance, and flow rates), optimal conditions were found for the formation of a stable Taylor cone and defect-free composite fibers. The morphology of the prepared electrospun composites was observed by scanning electron microscopy (SEM). The wettability, surface composition, and thermal and mechanical properties of the PMMA/TiO_2_ composites were studied in detail by water contact angle measurements, X-ray photoelectron spectroscopy (XPS), thermogravimetric analyses (TGA), and tensile tests. The optical properties, photocatalytic activity when using methylene blue as a model organic pollutant, and reusability of the composites were evaluated with respect to targeted application.

## 2. Materials and Methods

### 2.1. Materials

Poly(methyl methacrylate) (PMMA, average molecular weight ~350,000 g/mol) and titanium (IV) oxide (TiO_2_ nanopowder, 99.7% anatase) were purchased from Sigma Aldrich. *N*,*N*-dimethylformamide (DMF, p.a.) and methylene blue B (MB) were purchased from Merck. All reagents were of an analytical grade of purity and were used as received without further purification.

### 2.2. Electrospinning

Solutions of PMMA (15% *w*/*v*) in DMF were prepared by heating them at 50 °C using a reflux condenser. In order to obtain PMMA/5TiO_2_ and PMMA/10TiO_2_ composites, TiO_2_ nanopowder (5 and 10 wt. % with respect to PMMA) was added to the PMMA solutions. The obtained dispersions were homogenized by sonication for 1 h in an ultrasonic bath (Bandelin Sonorex, Berlin, Germany, 160/640 W, 35 kHz).

The experimental electrospinning setup comprised a high-voltage DC power supply, rotating collector, needle for syringe (i.d. 0.6 mm × o.d. 0.9 mm), and syringe pump (NE-300 New Era Pump Systems, Inc., Farmingdale, NY, USA) for delivering the spinning dispersions. The electrospinning of the PMMA solution and PMMA/TiO_2_ dispersions was performed at a 15 kV applied voltage with a needle tip-to-collector distance of 10 cm, a 2 mL/h flow rate, and a collector rotation speed of 1200 rpm. These optimal electrospinning conditions were identified through the testing of various combinations of processing parameters (applied voltage, flow rate, and needle tip-to-collector distance).

### 2.3. Characterization

The morphology of the electrospun PMMA/TiO_2_ composites was observed using scanning electron microscopy (SEM). Specimens were vacuum-coated with gold in a Jeol JFC-1200 fine coater and analyzed using a Jeol JSM-5510 instrument (JEOL Co., Ltd., Tokyo, Japan). The Image J software was used to measure the average fiber diameter by measuring at least 15 fibers from each image. Transmission electron spectroscopy (TEM) observations of the PMMA/TiO_2_ composites were carried out with a JEM-2100 instrument (JEOL Co., Ltd., Tokyo, Japan) equipped with selected area electron diffraction (SAED). Specimens were prepared by depositing on a copper grid. The surface chemical composition of the PMMA/TiO_2_ composites was determined quantitatively by X-ray photoelectron spectroscopy (XPS). The measurements were carried out in the UHV chamber of an ESCALAB-MkII (VG Scientific, Waltham, MA, USA) spectrometer using Mg Ka excitation with a total instrumental resolution of ~1 eV. Energy calibration was performed, taking the C1s line at 285 eV as a reference. Surface atomic concentrations were evaluated using Scofield’s ionization cross-sections with no corrections for λ (the mean free path of photoelectrons) and the analyzer transmission function. The experimental values for the element atomic percentages obtained from the XPS analysis were the average of three independent measurements. Water contact angles (WCA) were measured with a drop shape analyzer (DSA 20E, Krűss GmbH, Hamburg, Germany) at room temperature. Rectangular samples with a 15 mm width and 40 mm length were cut and fixed on glass slides (75 mm × 25 mm). Contact angles were measured before and after the UV light irradiation (UV lamp 400F/2, UVASPOT 400/T, Dr. Hönle AG, Gräfelfing, Germany) of the samples for 30 and 60 min. The distance from the UV lamp to the samples was 30 cm. A sessile drop of distilled water with a volume of 5 μL controlled by a computer dosing system was deposited onto the samples. The contact angles were determined by commercial software (DSA 20E, Krűss GmbH) using the acquired images of the droplet. The data were averages taken from 10 measurements for each sample.

X-ray diffraction (XRD) analyses were performed using a D8 Bruker Advance diffractometer with filtered Cu Kα radiation and a LynxEye detector at room temperature. XRD spectra were recorded in the 2θ range from 5.3° to 80° with a step size of 0.02° and counting time of 1 s/step. Phase identification was performed using the Diffracplus EVA using ICDD-PDF2 Database. Thermogravimetric analyses (TGA) were carried out on SetSys Evolution 2500 (Setaram, Caluire, France) at a heating rate of 10 °C/min and an argon flow of 30 mL/min. Emission spectra were recorded using a Cary Eclipse spectrofluorometer (Varian, Santa Clara, CA, USA). Rectangular specimens with a 10 mm width and 45 mm length were cut and their fluorescence spectra were recorded using quartz glass cells with 10 mm path length.

Tensile testing was performed at room temperature with a crosshead speed of 20 mm/min on a single column dynamometer INSTRON 3344 equipped with a 50 N load cell. Rectangular specimens with 20 mm widths and 60 mm lengths were cut in the collector rotation direction (0°). The thickness of each specimen was measured before the tensile test using a digital thickness gauge FD 50 (Käfer GmbH, Villingen-Schwenningen, Germany). The average values for the tensile strength (σ, MPa), elongation at break (ε_B_, %), and Young’s modulus (E, MPa) were determined based on the regression of the linear part of the stress–strain curves from at least 10 tested specimens of each electrospun material.

### 2.4. Photocatalytic Activity

The photocatalytic activity of the prepared electrospun materials was evaluated by the degradation of a model pollutant—methylene blue B (MB). A stock solution of MB (2 × 10^−5^ mol/L) in distilled water was prepared and used for a photodegradation analysis. Electrospun samples with a size of 10 mm × 10 mm were cut and then immersed in 10 mL of the MB solution. In order to reach an adsorption–desorption equilibrium, samples were kept in the dark for 60 min and were then irradiated by UV light (UV lamp 400F/2, UVASPOT 400/T, Dr. Hönle AG, Ilmenau, Germany) for 3 h. The photocatalytic degradation of MB was monitored spectrophotometrically by recording the intensity decrease in the MB absorbance at 660 nm with the irradiation time. UV–vis spectra were recorded with a DU800 spectrophotometer (Beckman Coulter Inc., Brea, CA, USA).

The reusability potential of the composed materials was also studied. For that purpose, after the first 3 h run the electrospun samples were again reused in two subsequent cycles by immersing them in a fresh MB solution. This procedure was repeated three times for 3 h using the same electrospun samples. After each cycle, the materials were washed and placed in fresh MB solution for the subsequent irradiation cycle. The photodegradation efficiency (PDE, %) was calculated from the following equation:

PDE (%) = (C_0_ − C_t_)/C_0_ × 100,

where C_0_ and C_t_ are the initial MB concentration and the concentration at time t, respectively.

## 3. Results and Discussion

The focus of the present work was on the fabrication of PMMA fibers filled with significant amounts of TiO_2_ and studying the effect of the inorganic photocatalyst on the properties of the obtained electrospun PMMA/TiO_2_ composites with respect to their targeted application. Koysuren et al. reported the synthesis of TiO_2_ particles by the sol–gel process, their calcination and immobilization in PMMA fibers by electrospinning [18]. However, the results show that the appropriate conditions for obtaining defect-free fibers have not been found, which calls into question the results of the research conducted. Moreover, the crystal phase of the synthesized TiO_2_ particles was not studied, and the presented FTIR spectra proved the presence of an unreacted titanium isopropoxide precursor. In addition, the amount of calcinated TiO_2_ particles incorporated into the PMMA fibers was very low, this is why only 20% of the methylene blue was degraded after 180 min of UV irradiation.

In the present study, in order to avoid all of these disadvantages, commercially available TiO_2_ (anatase) nanopowder was used. Furthermore, preliminary experiments were performed and by varying the electrospinning conditions (applied voltage, needle tip-to-collector distance and flow rates), optimal terms for the formation of defect-free composite PMMA fibers were found. Moreover, besides the electrospinning conditions, the TiO_2_ content in the dispersions was also varied toward the fabrication of PMMA fibers filled with a significant amount of TiO_2_. It was determined that the incorporation of the maximum amount of 10% of TiO_2_ nanopowder (with respect to PMMA) into the PMMA solution did not hamper the process of electrospinning and resulted in the preparation of continuous fibers. The effect of TiO_2_ on the properties of electrospun PMMA/TiO_2_ composites as a filler in PMMA fibers was studied in detail and the composition–structure–property dependence was determined.

### 3.1. PMMA/TiO_2_ Composites Fabrication and Characterization

Preliminary experiments showed that the electrospinning (15 kV applied voltage, 10 cm needle tip-to-collector distance, and 2 mL/h flow rate) of PMMA spinning solution in DMF with a concentration of 15% *w/v* resulted in defect-free PMMA fibers. These conditions were found to be suitable and were used for the fabrication of defect-free composite PMMA fibers containing 5 and 10% of TiO_2_.

**Figure 1:**
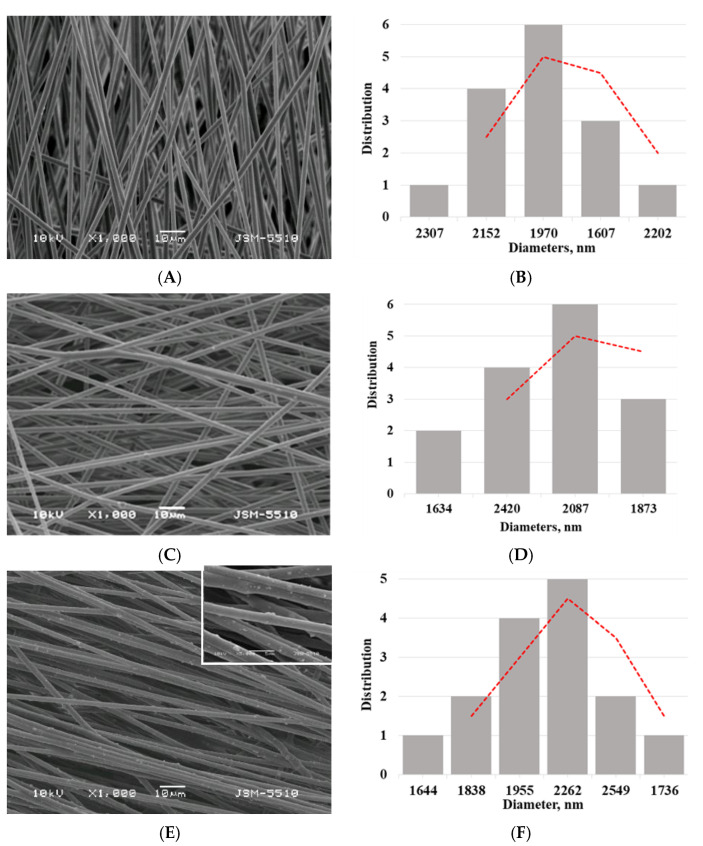
SEM micrographs and fibers diameter distribution of electrospun PMMA (**A**,**B**), PMMA/5TiO_2_ (**C**,**D**), and PMMA/10TiO_2_ (**E**,**F**) composites. Magnification ×1000. Inset: magnification ×5000.

The morphology of the fabricated PMMA/TiO_2_ composites was observed by SEM (Figure 1). The SEM micrographs clearly show that the selected concentrations and electrospinning conditions lead to the fabrication of cylindrical, uniform, and defect-free fibers with a certain fiber alignment in the direction of the collector rotation. Interestingly, the surface of the composite fibers was decorated with TiO_2_ (Figure 1C,E). Moreover, when the TiO_2_ concentration in the PMMA fibers was increased, the number of particles on the fiber surface increased (Figure 1E). This implies that part of the TiO_2_ loaded into the fibers aggregates during the electrospinning process and that the optimum photocatalyst concentration was reached, thus resulting in the particles being arranged not only in the PMMA fibers but also on their surface. The fiber diameter distribution is also presented in Figure 1. As expected, the TiO_2_ incorporation into the PMMA fibers resulted in a slight increase in their average fiber diameters—from 1986 ± 245 nm for PMMA fibers (Figure 1B) to 2079 ± 257 nm (Figure 1D) and 2104 ± 319 nm (Figure 1F) for PMMA/5TiO_2_ and PMMA/10TiO_2_, respectively. The SEM analyses clearly show that uniform and defect-free PMMA/TiO_2_ composite fibers were successfully fabricated in one step by electrospinning and that their morphologies depend on the TiO_2_ content in the PMMA fibers.

The successful inclusion of the TiO_2_ particles in the PMMA fibers was evidenced by TEM (Figure S1). As clearly seen, a part of TiO_2_ formed agglomerates during the electrospinning process. In confirmation of the SEM observations, TEM analyses also clearly showed that the surface of the composite fibers was decorated with TiO_2_. In addition, the obtained SAED patterns showed strong diffraction spots evidencing the lattice spacing of the crystals, indexed to the anatase structure of TiO_2_ (# 96-101-0943) with the lattice planes.

**Figure 2:**
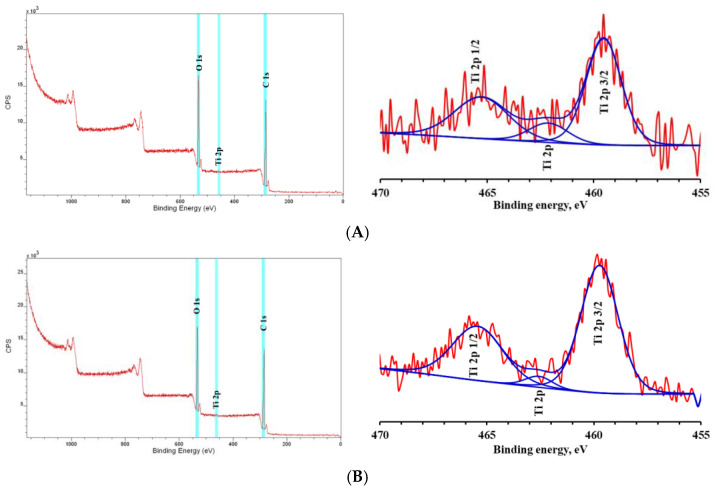
Survey XPS (left, bands for C1s, O1s, and Ti2p are marked in blue) and Ti2p (right, splitting is marked with blue lines) spectra of electrospun PMMA/5TiO_2_ (**A**) and PMMA/10TiO_2_ (**B**) composites.

The surface chemical composition of the PMMA/TiO_2_ fibers was evaluated by XPS analysis (Figure 2) and provided additional evidence regarding the titanium content on the fibers’ surfaces. In the survey of the XPS spectra of PMMA/TiO_2_ composite fibers, photoemission bands corresponding to C1s (285 eV), O1s (532.4 eV), and Ti2p (459 eV) were observed (Figure 2, left). As expected, in the Ti2p spectrum of PMMA/TiO_2_ composites, one major peak was observed at 459 eV with a splitting of 5.7 eV for the Ti2p 3/2 and Ti2p 1/2 components of the doublet (Figure 2, right). The binding energy value is characteristic of titanium in its normal Ti^4+^ oxidation state and is in good agreement with the value reported in the literature [22]. By calculating the Ti/C atomic ratio, it was determined that the titanium content on the surface of PMMA/5TiO_2_ (0.47%) was lower than that on the PMMA/10TiO_2_ (0.82%) composites. Therefore, the XPS analysis further verified that the surface composition of the electrospun PMMA/TiO_2_ composites was affected by the amount of TiO_2_ incorporated in the PMMA fibers.

**Figure 3:**
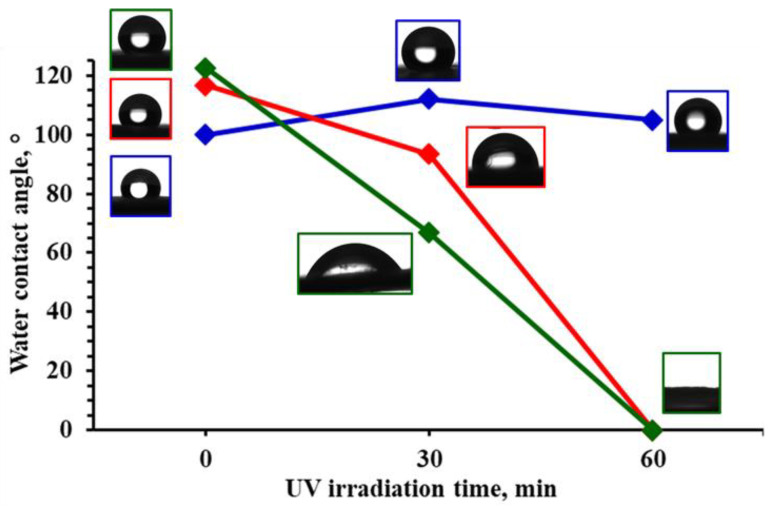
Water contact angles and digital images of water droplets on electrospun PMMA (blue), PMMA/5TiO_2_ (red), and PMMA/10TiO_2_ (green) composites before and after UV irradiation.

The possibility of altering the surface wettability of the PMMA/TiO_2_ composites was particularly crucial from a practical point of view. It is well known that UV light treatment enhances the wettability of different titanium surfaces [7,23]. SEM and XPS analyses unambiguously proved the presence of TiO_2_ on the composites’ surfaces. For that reason, the water contact angles were measured before and after UV light irradiation in order to study the alteration in surface wettability of the PMMA/TiO_2_ composites. As seen in Figure 3, before UV irradiation the PMMA fibers showed water contact angles of 111.5° ± 5.17° due to their hydrophobic behavior [24]. As expected, the addition of TiO_2_ led to the increase in contact angles to 116.7° ± 3.75° and 122.6° ± 4.24° for PMMA/5TiO_2_ and PMMA/10TiO_2_, respectively. These results were in accordance with our previous findings and with the literature data [25,26]. Therefore, before UV irradiation the contact angles were in the hydrophobic region (above 90°), which is due to the hydrophobic surface of the PMMA fibers. The presence of TiO_2_ on the surfaces of the composites imparted additional hydrophobicity to the PMMA/TiO_2_ composites. After 30 min of UV irradiation (Figure 3), the PMMA fibers remained hydrophobic and showed a water contact angle of 112° ± 4.15°. In contrast, the contact angles of PMMA/5TiO_2_ and PMMA/10TiO_2_ composites decreased significantly to 93.5° ± 3.46° and 66.9° ± 2.36°, respectively. It is noteworthy, however, that after 60 min of UV irradiation (Figure 3) only PMMA fibers preserved their hydrophobicity with a contact angle of 109.8° ± 3.60°, while the contact angle for both PMMA/5TiO_2_ and PMMA/10TiO_2_ was 0°. Thus, the change in the wettability of PMMA/TiO_2_ composites strongly depends on the fiber surface composition, and a highly hydrophilic surface might be achieved by UV irradiation.

**Figure 4:**
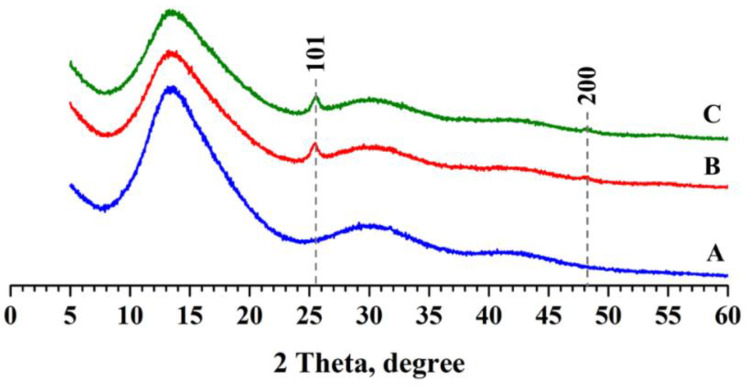
XRD patterns of electrospun PMMA (A), PMMA/5TiO_2_ (B), and PMMA/10TiO_2_ (C) composites.

The crystal phase of TiO_2_ in the composite fibers was identified by X-ray diffraction (XRD) and compared with those of the PMMA fibers (Figure 4). The diffraction pattern of the PMMA fibers showed a broad diffraction plane at 2θ = 13.7° (intensive), as well at 2θ = 30.0° and 42.7° (lower intensities), characteristic of its amorphous structure. In addition to these planes of PMMA, the XRD patterns of PMMA/TiO_2_ composites exhibited characteristic TiO_2_ diffraction peaks at 2θ = 25.6° (101) and 48.2° (200) (Figure 4) for the anatase crystal phase. These results indicated that the TiO_2_ incorporated in the PMMA fibers did not affect the structure of PMMA and that these components did not react during the electrospinning. The obtained results are in agreement with the TiO_2_ (anatase) standard data (ICDD-PDF2 #00-021-1272).

**Figure 5:**
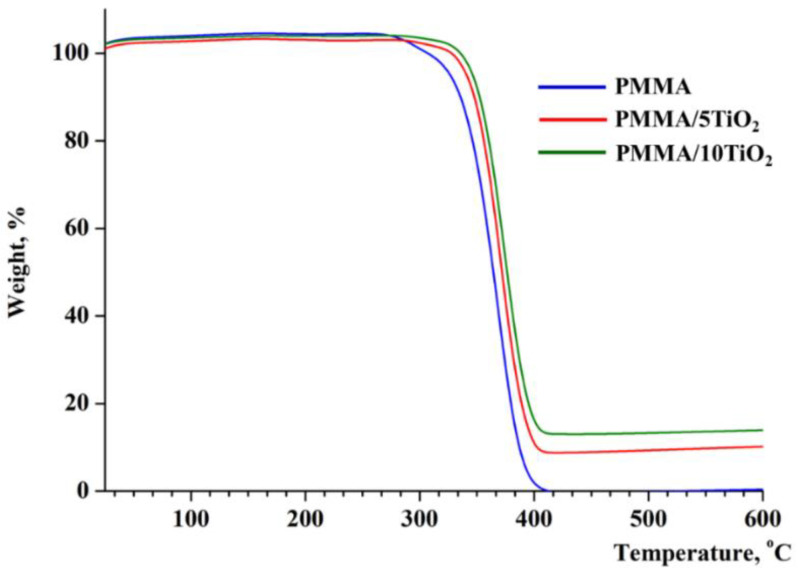
TG curves of electrospun PMMA, PMMA/5TiO_2_, and PMMA/10TiO_2_ composites.

The effect of TiO_2_ content on the thermal stability of the PMMA/TiO_2_ composites was studied by TGA (Figure 5). Apparently, the thermal decomposition of PMMA and composites began at 300 °C and ended at 400 °C due to the decomposition of PMMA. As can be seen, the thermal stability of the composites was slightly shifted to a higher decomposition temperature—373 °C for PMMA/5TiO_2_ and 375 °C for PMMA/10TiO_2_—in comparison to that of PMMA fibers (369 °C). These results indicated that the addition of TiO_2_ slightly increased the thermal stability of the obtained composites. It is notable that in thermal decomposition of the PMMA/5TiO_2_ and PMMA/10TiO_2_ composites, the presence of 5.3% and 10.9% residues was observed (Figure 5, red and green lines). These residues are due to the inorganic TiO_2_ component, which did not undergo any alteration at these temperatures. These weight losses were very close to the initial TiO_2_ amounts added to the PMMA solutions.

**Figure 6:**
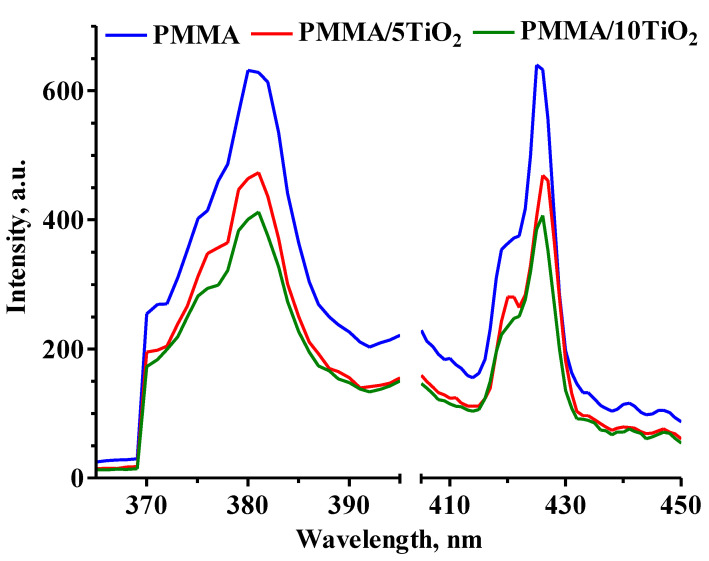
Excitation spectra (**left**) and the corresponding emission spectra (**right**) of electrospun PMMA (blue line), PMMA/5TiO_2_ (red line), and PMMA/10TiO_2_ (green line) membranes.

In order to study the effect of the TiO_2_ content on the optical properties of PMMA, the excitation and emission spectra of electrospun materials were recorded and compared (Figure 6). The excitation spectrum of PMMA fibers had a maximum at 380 nm, which corresponds to the absorption maximum of the polymer, while its fluorescence maximum was at 425 nm. Interestingly, the fluorescence intensity was quenched upon the addition of TiO_2_, and this quenching depended on the amount of TiO_2_ added. The same positions of the maxima in the spectra indicate that TiO_2_ did not change the structure of the PMMA matrix. On the other hand, the decrease in the fluorescence intensity can be explained by the interaction of TiO_2_ with the lone pairs of oxygen atoms in the structure of PMMA, which led to a change in its polarization.

### 3.2. Tensile Properties of the PMMA/TiO_2_ Composites

In view of the potential application of these composites, we decided to study their tensile strength and compare the values obtained with those of PMMA. A serious problem in the accurate evaluation of the mechanical properties of electrospun materials is the lack of standardization in conducting tensile tests. Recently, we showed that specimens cut in the direction of the collector rotation (0°) have better mechanical properties [27]. For that reason, this already developed standardized methodology was applied for the tensile testing of the electrospun composites. The average thickness of all specimens was ~0.312 mm.

**Figure 7:**
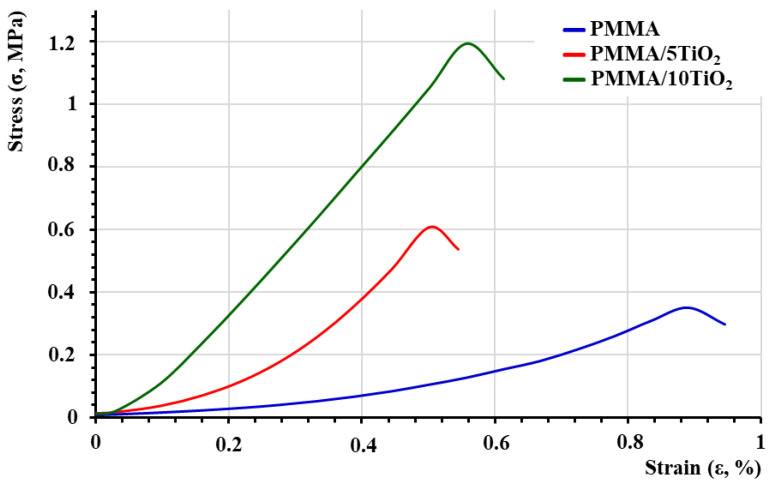
Stress–strain curves of electrospun PMMA (blue line), PMMA/5TiO_2_ (red line), and PMMA/10TiO_2_ (green line) composites.

Stress–strain (σ/ɛ) curves performed at tension of specimens at a constant rate are shown in Figure 7. All curves showed a smooth linear increase in the stress with increasing strain. The curve for PMMA fibers displayed a high deformation at relatively low stress. As can be seen, the composite fibers had better mechanical properties compared to the PMMA fibers. Moreover, the addition of TiO_2_ resulted in a proportional increase in the stress with increasing strain, which ended with the sample breaking.

From the stress–strain curves, the values of the modulus of elasticity (E, MPa), tensile strength (σ, MPa), and elongation at break (ε_B_, %) were determined; these are presented in Table 1. It is obvious that the composite materials had a higher modulus of elasticity and tensile strength compared to the PMMA fibers. Furthermore, the tensile strength of the composites increased almost twice as the TiO_2_ amount increasing. In contrary, the elongation at break of the PMMA fibers was more than three times greater than that of the composites. These results indicate that the addition of TiO_2_ improved the elasticity of the composites and contributed to reducing their brittleness. In addition, the composites provided a higher loading.

### 3.3. Photocatalytic Degradation of MB and Reusability of PMMA/TiO_2_ Composites

**Figure 8:**
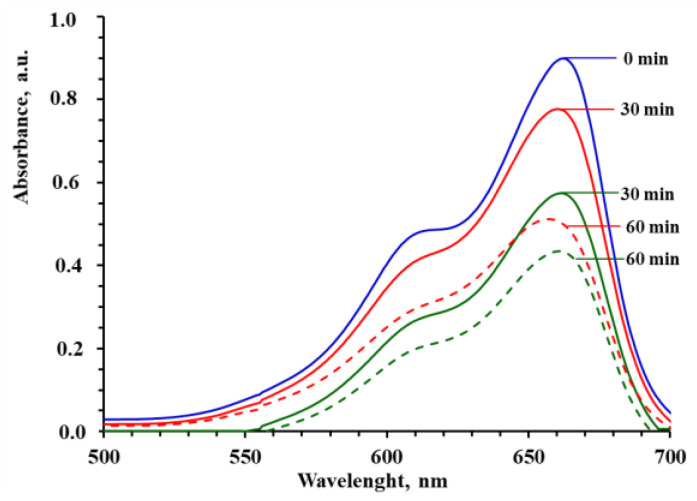
Absorption spectra of MB (blue line) after UV light irradiation (30 and 60 min) in the presence of electrospun PMMA/5TiO_2_ (red lines) and PMMA/10TiO_2_ (green lines) composites.

The photocatalytic activity of the composites was studied in the presence of the model organic pollutant—methylene blue (MB)—and monitored spectrophotometrically at 660 nm. The obtained results show a decrease in MB absorption as a function of irradiation time (Figure 8). It can be seen that PMMA/5TiO_2_ composites degrade ca. 20% of the dye after 30 min of irradiation, while after 60 min they degrade approximately 50%. Clearly, in the presence of PMMA/10TiO_2_ composites, 50% of the dye degraded after 30 min—i.e., composites containing large amounts of TiO_2_ were more effective. This effect was expected, because the amount of TiO_2_ particles is higher on the surface of PMMA/10TiO_2_ composites, which accelerates the photocatalytic action.

**Scheme 1:**
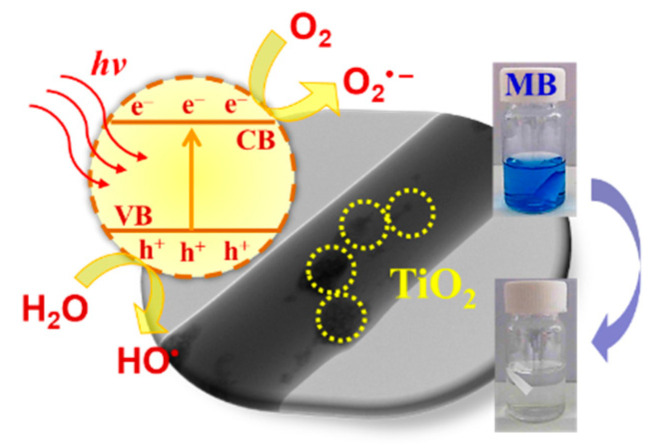
Representation of the photocatalytic degradation of MB in the presence of PMMA/TiO_2_ composites.

Schematic representation of the photocatalytic degradation of MB in the presence of PMMA/TiO_2_ composites is shown in Scheme 1.

**Figure 9:**
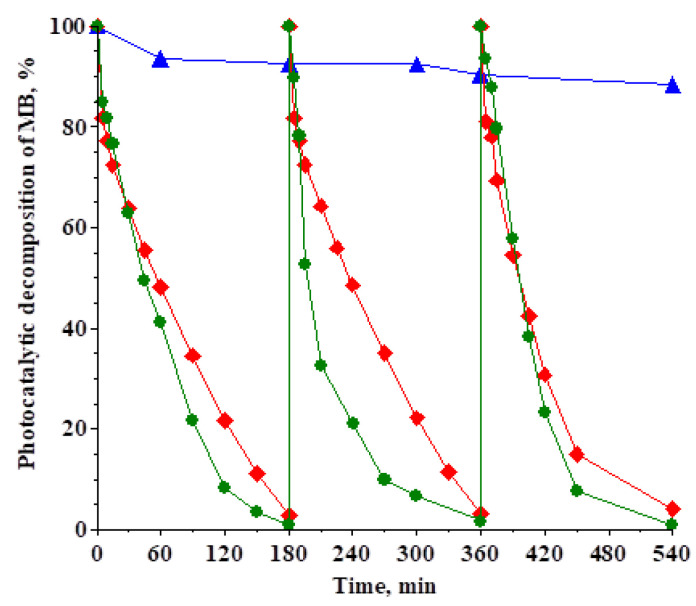
Three consecutive cycles of MB degradation at 660 nm under UV light irradiation in the presence of electrospun PMMA (blue line), PMMA/5TiO_2_ (red line), and PMMA/10TiO_2_ (green line) composites.

The photocatalytic activity of the composites during three irradiation cycles was also measured in order to evaluate their activity during repeated use (Figure 9). The reaction of a blank sample (PMMA fibers) was monitored as well. As can be seen, in the presence of PMMA fibers, MB preserved its stability and a very slight discoloration under UV irradiation was observed (Figure 9, blue line). After 9 h of irradiation, the calculated photodegradation efficiency was only 11.5%. Thus, the photocatalytic activity of the composites was determined to be due to the embedded TiO_2_. Undoubtedly, the composites exhibited excellent stability—at the end of the third cycle, the calculated PDEs were 95.8% and 99.1% in the presence of PMMA/5TiO_2_ and PMMA/10TiO_2_, respectively. These results prove that the composites maintain their photocatalytic activity even after three uses.

## 4. Conclusions

In the present study, PMMA/TiO_2_ composites were successfully fabricated in one step by electrospinning. Besides the electrospinning conditions, the TiO_2_ content in the PMMA solution was varied in order to fabricate PMMA fibers filled with significant amounts of TiO_2_. It was shown that the incorporation of a maximum of 10% TiO_2_ into the PMMA solution did not hamper the electrospinning and resulted in the preparation of continuous and defect-free composite fibers. Clearly, the morphology and surface chemical composition of electrospun PMMA/TiO_2_ fibers depends on the TiO_2_ content. Contact angle measurements revealed that the wettability of the composites might be easily changed from hydrophobic to superhydrophilic after 1 h of UV light irradiation. In particular, TiO_2_ had a slight impact on the thermal stability and optical properties of the composites. However, the incorporation of the inorganic component resulted in a significant increase in the modulus of elasticity and tensile strength and a decrease in elongation at break. Furthermore, PMMA/TiO_2_ composites preserve their photocatalytic activity almost completely after three uses in the presence of a model organic pollutant. Thus, the proposed original and simple approach is very promising for the future development of highly efficient membranes for the degradation of organic pollutants.

## Figures and Tables

**Table 1:** The mechanical characteristics of electrospun materials.

Samples	Young’s Modulus (E, MPa)	Tensile Strength (σ, MPa)	Elongation at Break (ε_B_, %)
PMMA	82.4	0.50	1.95
PMMA/5TiO_2_	237.4	0.61	0.50
PMMA/10TiO_2_	251.9	1.19	0.56

## Data Availability

The data presented in this study are available on request from the corresponding author.

## Supplementary Materials

The following are available online at https://www.mdpi.com/2073-4360/13/22/3923/s1, Figure S1: TEM micrograph and SAED patterns of the PMMA/5TiO_2_ composites.
