# Supplementary material for: Electrospun Poly(methyl methacrylate)/TiO2 Composites for Photocatalytic Water Treatment
Source: Polymers (Basel). 2021 Nov 13;13(22):3923. doi: 10.3390/polym13223923 (PMC8617697; doi:10.3390/polym13223923)
Supplement: Supplementary file 1 [file polymers-13-03923-s001.zip › polymers-1456798-supplementary.pdf]

polymers

MDPI

Supplementary material

Electrospun Poly(methyl methacrylate)/TiO_2 Composites for Photocatalytic Water Treatment

Olya Stoilova *, Nevena Manolova, and Iliya Rashkov

Laboratory of Bioactive Polymers, Institute of Polymers, Bulgarian Academy of Sciences, Acad. G. Bonchev St., bl. 103A, BG-1113 Sofia, Bulgaria; stoilova@polymer.bas.bg; manolova@polymer.bas.bg (N.M.); rashkov@polymer.bas.bg (I.R.)

* Correspondence stoilova@polymer.bas.bg

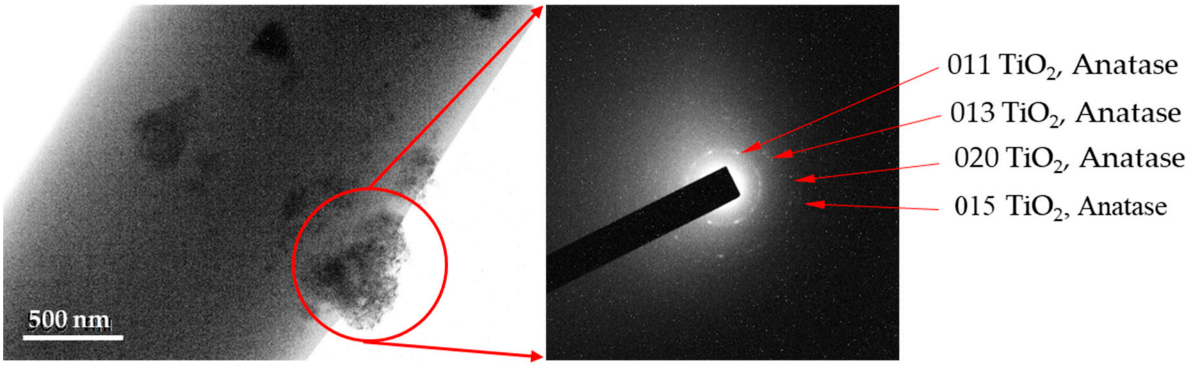

Figure S1. TEM micrograph and SAED patterns of the PMMA/5TiO_2 composites.
